# Effect of Topical Estrogen in the Mangement of Traumatic Facial Wounds 

**Published:** 2016-01

**Authors:** Seyed Amirhosein Ghazizadeh Hashemi, Behrooz Barati, Hosein Mohammadi, Masumeh Saeidi, Abbas Bahreini, Mohammad Ali Kiani

**Affiliations:** 1 *Department of Otorhinolaryngology, Shahid Beheshti University of Medical Sciences, Tehran, Iran.*; 2 *Students Research Committee, Faculty of Medicine, Mashhad University of Medical Sciences, Mashhad, Iran.*; 3 *Students Research Committee, Faculty of Medicine, Shiraz University of Medical Sciences, Shiraz, Iran.*; 4 *Department of Pediatrics, Mashhad University of Medical Sciences, Mashhad, Iran.*

**Keywords:** Estrogen, Effect, Traumatic Wounds, Treatment

## Abstract

**Introduction:**

Acute skin wound healing is a complicated process comprising various phases. Recent animal studies have shown that steroid sex hormones such as estrogen maybe helpful in the regulation of several pathophysiologic stages that are involved in wound healing. In this study we examined the effects of topical estrogen in the treatment of traumatic facial wounds.

**Materials and Methods:**

Patients referred to Luqman Hospital, Tehran with traumatic wounds were enrolled in this case-control study into two groups of equal size. From the second week of the study, topical estrogen (0.625 mg conjugated topical estrogen ointment) was administered in the case group, while the control group received a Eucerin dressing only. The two groups were then compared in terms of wound healing rate on Day 7,14, and 30.

**Results:**

Thirty patients with mean age of 16.02+36.23 years were compared in the control and estrogen-treated groups. After treatment, no scars or keloids were observed in either group. The wound area in the estrogen group was lower than that in the control group on Day 14 and 30, but the difference was not significant (P>0.05). Healing rates in the control group on Day 14 (7.1+42.3 vs.50.3+4.9 mm2) and Day 30 (1.9+93.5 vs. + 97.3+0.6 mm2) (were lower than those in the estrogen group, but the differences were not significant (P>0.05). Findings show that the required time for wound healing in the estrogen-treated group was lower than that in the control group, but the difference was not significant (P>0.05).

**Conclusion:**

Based on this study, topical estrogen has no effect on the rate of wound healing or the rate of wound area.

## Introduction

Skin is the largest organ of the body and a physical barrier between the body and the surrounding environment. Maintaining the integrity of the skin in humans and animals is of the utmost importance, because protects them against dehydration, bleeding and invasion of microorganisms (1). The term ‘wound’ refers to the loss of epidermis and dermis. Wounds are caused following skin injury, and may be classified as open or closed. Several local and systemic factors, such as age, medication, nutritional status, circulation, tissue hypoxia, use of topical components and wound dressing, have an effect on wound healing. Prolonged healing times mean that the wound may become prone to bacterial infections and an increased probability of forming disfiguring scars leading to physical, emotional and mental problems for the patient. Therefore, recently, the use of agents that decrease wound healing time have attracted the attention of medical researchers. The antiepileptic drug, oral phenytoin, has been used over the past 60 years, and is known to cause gingival hypertrophy following long-term use. Because oral phenytoin increases angiogenesis and increases tensile strength in wound healing, it has been studied in several trials to investigate its potential in a number of types of wound (1-4).

In a study of ovariectomized mice, Gal et al., showed that treatment with topical estradiol causes angiogenesis and greater collagen precipitation in wounds. Systemic or local administration of systemic or topical 17-beta-estradiol in menopausal women has also been shown to result in acceleration in the wound healing process (5). In a review published in 2007, it was shown that a reduction in wound healing is common in post-menopausal women and many of the reviewed studies showed that estrogen improves the healing process in these women. This review also showed that androgens cause a delay in healingand interfere with the protein aggregation that

leads to healing (6). 

Agglutination or wound healing is a recovery process that is caused following lesions of the skin and other tissues. One of the goals of medicine is to achieve faster wound healing with fewer side effects. Reduction of wound healing time is of particular importance in order to minimize possibility of infection or wound complications and to reduce costs. Previous studies have principally examined the molecular aspects of this issue. The objective of this study was to determine the effects of topical estrogen on the treatment of traumatic facial wounds in patients referred to the ear, nose and throat (ENT) clinic of Luqman Hospital, Tehran from 2012 to 2013. 

## Materials and Methods

In this clinical trial, conducted between February 2012 and August 2013, patients with traumatic wounds who had been referred to the ear, nose, and throat (ENT) clinic at Luqman Hospital in Tehran, Iran, were enrolled in two groups (n=15 in each group). One group was treated with topical estrogen (0.625 mg conjugated topical estrogen cream), while in the other group a simple dressing only was used (Eucerin-treated). Estrogen and Eucerin were administered from the second week of the study. Wound healing was monitored through the use and evaluation of photographs of patients at the same stages of the study.

For all 30 patients, wounds were evaluated and photos were taken at specified periods (on Day 1,7, and 14), and compared against the first day of treatment. Wound area (mm2) and healing rate (days) in the estrogen and Eucerin groups were compared on Days 7, 14, and 30. Data were entered into SPSS software version 21, and the two groups were compared using a t-test and covariance and the Mann-Whitney test. A p-value less than 0.05 was considered significant. 

## Results

Thirty patients with mean age of 16.02+36.23 years in matched estrogen- and Eucerin-treated groups were compared. No scars or keloids were reported in any case. Wound area was examined on Day 7, 14, and 30. Wound area in the estrogen group on Days 14 and 30 was lower than that in the Eucerin group, but this difference was not significant (P>0.05) (Table.1).

**Table 1:** Mean ±standard deviation wound area in the estrogen and Eucerin groups on Day 7,14, and 30 (mm^2^)

**Variables**	**Day 7**	**Day 14**	**Day 30**
Eucerin group (N=15)	296.9+15.8	141.8+23.7	21.10+7.20
Estrogen group (N=15)	260.9+26.3	125.3+16.1	6.20+1.5
P-value	P>0.05	P>0.05	P>0.05
			

Healing rate in the Eucerin group was lower than that in the estrogen group on Day 14 and 30, but this difference was not significant (P> 0.05) (Table.2).

**Table 2:** Wound healing rate in the Eucerin and estrogen groups on Day 14 and 30 (%).

**Variables**	**Day 14**	**Day 30**
Eucerin group (N=15)	42.3+7.1	93.5+1.9
Estrogen group (N=15)	50.3+4.9	97.3+0.6
P-value	P>0.05	P>0.05

Time required for wound healing in the estrogen-treated group was lower than that in the Eucerin-treated group, but this difference was not significant (P>0.05). 

## Discussion

Agglutination is a healing process triggered following the formation of lesions of the skin and other soft tissue and consists of a series of physiological and biochemical events in the cells of all organisms. Studies of the effect of estrogen on wound healing have been reported, but most studies have been performed in mice. Some studies have been carried out to examine the effect of wound healing in post-menopausal women in comparison with pre-menopausal women who have sufficient estrogen. In addition to its effects on the reproductive system, estrogen has important effects on angiogenesis, proliferation and the growth of cells and increases the secretion of growth hormone. Between men and women, and also between pre- and post-menopausal women, there is a distinct difference in wound healing that shows the effect of sex hormones on the wound healing process. Estrogen accelerates wound healing, while androgens have an inhibitory effect on wound healing. A lack of systemic hormones in older women due to the menopause leads to a delay in healing of skin wounds that can be ameliorated by local and systemic estrogen replacement (6-8). A review by Ashcroft et al. showed that a reduction in wound healing is common in post-menopausal women and administration of estrogen improves the recovery process (6). Other studies on animals have shown that castration achieves an increase in the recovery rate, while testosterone inhibits recovery (9,10).

A study by Giliver et al. on mice showed that 5-alpha-redutase antagonists increase the rate of wound healing in patients (9). Another study by the same author, showed that estrogen has a positive effect on wound healing and that androgens have an inhibitory effect (11). Several studies reported in 2003 showed that an increase in testosterone levels has a negative effect on wound healing, whereas in the same year, Labrie et al. demonstrated the positive effect of testosterone in wound healing (10,12-15). In 2003, Abe et al. showed that estrogen directly or indirectly increases the production of Macrophage migration inhibitory factor in the regulatory T cell which is one of the most important mediators in wound healing. This factor has an important role in the regulation of inflammation and innate and acquired immunity, and estrogen accelerates wound healing by increasing its production (16). Results of a study by Ashcroft et al., in which topical estrogen was administered by injection into patients, showed significant wound healing (17). Khaksar et al. published reports of the role of estrogen in wound healing in 2011 (18).

In this study we worked with human samples, unlike many previously published articles. The results of this study showed that the wound area in the estrogen group was lower than in the Eucerin group on Day 14 and 30, but that this difference was not statistically significant. Most studies on mice have shown that estrogen has a positive effect on wound healing in patients (19,20). In the present study we showed that estrogen shortens the healing time, but the difference was not statistically significant between groups. The results of the study by Mir nezami et al. showed that estrogen shortens the duration of wound healing (20). Results of our study also showed that the recovery rate in the Eucerin group was lower than in the estrogen group. However, these differences were not statistically significant.

In the present study, no scars or keloids were reported in any patients in either the estrogen-treated group or the Eucerin-treated group during a 1–year follow-up. Furthermore, infection and inflammation were not observed in any of the treated patients in either group. Formation of keloids and scars is one major problem associated with wound healing which causes subsequent health problems, repeated visits and psychological distress for the patient. Hypertrophic scars and keloids are a form of intensified conciliation response, and their treatment is challenging for clinicians. However, if keloids are formed, their treatment will be difficult and regardless of the treatment used, there will be a high flare-up rate (21).

## Conclusion

The effect of Eucerin and estrogen on wound healing and the time required for healing of facial wounds was almost equivalent, and both drugs studied prevented the formation of scars and keloids on facial wounds. In other words, topical estrogen has no effect on the rate of wound healing or the rate of wound area . 
